# Similar cost-utility for double- and single-bundle techniques in ACL reconstruction

**DOI:** 10.1007/s00167-017-4725-1

**Published:** 2017-09-22

**Authors:** N. Sernert, E. Hansson

**Affiliations:** 1 Department of Research and Development, NU Hospital Group, Trollhättan, Sweden; 2 Institute of Health and Care Sciences, Sahlgrenska Academy, Gothenburg, Sweden; 3 Institute of Clinical Sciences, Gothenburg University, Gothenburg, Sweden; 4 Department of Orthopaedics, Sahlgrenska University Hospital, Gothenburg, Sweden

**Keywords:** HRQoL, QALY, ICER, Sensitivity analyses, ACL injury, Single- and double-bundle technique

## Abstract

**Purpose:**

The aim was to estimate the cost-utility of the DB technique (*n* = 53) compared with the SB (*n* = 50) technique 2 years after ACL reconstruction.

**Methods:**

One hundred and five patients with an ACL injury were randomised to either the Double-bundle (DB) or the Single-bundle (SB) technique. One hundred and three patients (SBG *n* = 50, DBG *n* = 53) attended the 2-year follow-up examination. The mean age was 27.5 (8.4) years in the SBG and 30.1 (9.1) years in the DBG. The cost per quality-adjusted life years (QALYs) was used as the primary outcome. Direct costs were the cost of health care, in this case outpatient procedures. Indirect costs are costs related to reduce work ability for health reasons. The cost-utility analysis was measured in terms of QALY gained.

**Results:**

The groups were comparable in terms of clinical outcome. Operating room time was statistically significantly longer in the DBG (*p* = 0.001), making the direct costs statistically significantly higher in the DBG (*p* = 0.005). There was no significant difference in QALYs between groups. In the cost-effectiveness plane, the mean difference in costs and QALYs from the trial data using 1000 bootstrap replicates in order to visualise the uncertainty associated with the mean incremental cost-effectiveness ratio (ICER) estimate showed that the ICERs were spread out over all quadrants. The cost-effectiveness acceptability curve showed that there was a 50% probability of the DB being cost-effective at a threshold of Euro 50,000.

**Conclusion:**

The principal findings are that the DB is more expensive from a health-care perspective. This suggests that the physician may choose individualised treatment to match the patients’ expectations and requirements.

## Introduction

Anterior cruciate ligament (ACL) rupture is an injury that is mostly related to sports, especially contact sports. However, the consequences affect not only the person’s opportunity to continue with his/her sporting activities, perhaps resulting in a future career being ruined. It also affects the whole life, as several professions, such as heavy engineering work and construction work, require stable knees. At a time when health-care costs and the effectiveness of health treatments are major topics for discussion, every orthopaedic department has to consider the most cost-effective surgery in order to prioritise and provide the maximum additional effects per additional unit of resource consumed. According to the Swedish ACL Register, an injury frequency of approximately 80 per 100,000 inhabitants in Sweden would mean that some 5800 individuals suffer anterior cruciate ligament injuries every year and that some 3500 undergo surgery [16].

Finding the optimal surgical technique is of great interest to every anterior cruciate ligament (ACL) surgeon. In the last decade, the double-bundle (DB) technique was introduced by restoring both the antero-medial and postero-lateral bundles.

The question of whether the DB technique is able to restore rotational instability to a higher degree compared with the single-bundle (SB) technique is still the subject of debate. The findings in recent systematic reviews are in favour of the DB technique in terms of rotational laxity and IKDC grade [22, 37, 38].

However, the DB technique is also more demanding and time-consuming and requires two fixation devices, thereby increasing the cost of the whole procedure. According to a meta-analysis, treatment with the DB technique is in favour in terms of knee joint stability and knee joint function [27] but, at least from a health-care perspective, more costly than the SB technique. This is in line with Paxton et al., who concluded that the double-bundle technique may be cost-effective in terms of IKDC grade [33]. In 2009, Brophy et al. [9] compared the cost of the SB and DB techniques and concluded that DB added considerable costs to the health-care system with small clinical benefits to patients. Comparing health-related quality of life, Nunez et al. [31] reported no significant difference between the SB and DB techniques, but concluded that the SB technique was more cost-effective in terms of knee joint stability and knee joint function.

Another aspect that should be considered when comparing the two techniques is whether the new technique could shorten sick leave—in other words, whether the new technique will offer a more stable knee earlier in the rehabilitation process, resulting in a shorter period of sick leave and thereby reducing the societal costs [18]. The social insurance system in Sweden is financed through social insurance contributions, and the individual has a legal right to compensation due to lost income (http://www.riksdagen.se/sv/Dokument-Lagar/Lagar/Svenskforfattningssamling/Socialforsakringsbalk-201011_sfs-2010-110/).

The available knowledge could form the basis of a cost-utility analysis (CUA), where the expected benefits are related to the costs. The result of a CUA is presented as cost per quality-adjusted life year (QALY). The CUA result can be used by decision-makers when deciding which health-care interventions should be made available in collectively funded health-care systems [7].

Clinical research often involves comparing the clinical outcome of the new and old technique. However, it is important to relate the efficacy of the new treatment to the costs [10], and many studies evaluate the cost-effectiveness from a health-care perspective (only including costs of health care); in the present study a societal perspective was used, including all costs involved after an ACL reconstruction.

The aim of the present study was to estimate the cost-utility of the DB technique when compared with the SB technique in patients after ACL reconstruction. The hypothesis was that the choice of surgical technique affects the cost-utility.

## Materials and methods

Data for the economic evaluation were collected alongside a randomised controlled study comparing the DB and SB techniques in 105 patients with ACL injuries 2 years after surgery. A CUA was conducted based on a comparison of the two surgical techniques. Prevalence-based direct and indirect costs were calculated from a societal perspective and compared to quality-adjusted life years estimated using the Euro QoL group five dimensions questionnaire for health-related quality of life (EQ-5D).

### Design

**Fig. 1:**
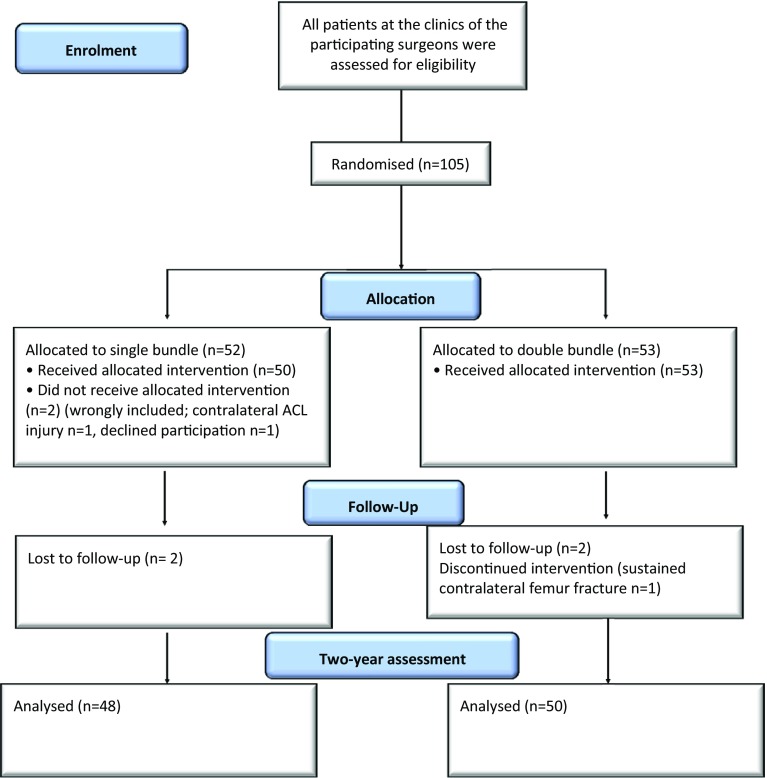
Flow chart according to consort of included patients. Only patients with complete data on costs and effects (utility) (baseline and at three months) were included in the CUA

The study was conducted between 2008 and 2009 at two different hospitals in the western part of Sweden (Fig. 1). Patient-specific resources used and preference-based health status data were collected on an individual basis. A complete description of the study design and results of the clinical intervention have been published previously [1].

The cost difference was calculated by subtracting the mean cost of DB from the mean cost of SB. The cost per quality-adjusted life year (QALYs) was used as the primary outcome. The difference in QALYs was calculated by subtracting the mean QALYs for DB from the mean QALYs for SB. The economic evaluation was performed from a societal perspective.

### Study cohort

The inclusion criterion was unilateral ACL injury in subjects over 18 years of age. The exclusion criteria were posterior cruciate ligament injury, more than 1+ medial or lateral collateral ligament laxity after injury or major previous knee surgery. Patients who fulfilled the inclusion criterion were consecutively asked to participate in the study. The participants were randomised to either the DB or the SB technique using closed envelopes administered by the same study coordinator for both hospitals.

At the 2-year follow-up, 103 patients answered the EQ-5D questionnaire on health-related quality of life. The single-bundle group (SBG) consisted of 50 patients, 35 males and 15 females, while the double-bundle group (DBG) consisted of 53 patients, 35 males and 18 females. The mean age in the SBG was 27.5 (8.4) years, whereas it was 30.1 (9.1) years in the DBG. Of these, 98 patients, 49 in each group, attended a clinical examination 26 (3.0) months after the index operation (Table 1, Fig. 1).

**Table 1:** Descriptive data

	Double bundle (DB)	Single bundle (SB)
Number of patients	53	50
Gender male/female	35/18	35/15
Age (years)		
Mean (SD)	30.1 (9.1)	27.5 (8.4)
Median (range)	29.0 (18-52)	25 (18-51)
Profession (%)		
Student	8 (15.1)	12 (24)
Labourer	27 (50.9)	26 (52)
Clerical	17 (32.1)	9 (18)
Unemployed		2 (4)
Missing values	1 (1.9)	1 (2)
Follow-up period [months; mean (SD)]	25.5 (3.5)	25.6 (2.3)
Missing values	1	3
Pre-injury Tegner activity level		
Mean (SD)	7.3 (1.5)	7.8 (1.3)
Median (range)	8 (5–9)	8 (3–9)
Missing value	2	
Time between the injury and index operation (months)	9 (2–240)	10 (3–240)

Two hospitals were involved in the present study; two experienced surgeons at each hospital performed all the ACL reconstructions using the same techniques. The learning curve was similar for all four surgeons. At Hospital A, 31 ACL reconstructions were performed (17 SB and 14 DB), and at Hospital B, 71 (33 SB and 38 DB) ACL reconstructions were performed.

The overall clinical outcome presented in Ahldén et al. [1] was that the clinical assessments at follow-up revealed no significant differences between the DB and SB groups in terms of knee laxity measurements, range of motion, Lysholm knee scoring scale, Tegner activity scale, Knee Injury and Osteoarthritis and Outcome Score (KOOS) or functional tests. A significant improvement was seen in both groups compared with the pre-operative values in terms of most clinical assessments.

### Surgical method

Standard antero-lateral and antero-medial portals were established. Associated intra-articular injuries, such as meniscal injuries and chondral lesions, were addressed at the time of the index operation.

The surgical procedure for both the single- and double-bundle techniques is described in detail in the article by Ahldén et al. [1].

#### Post-operative procedure

All patients had their surgery performed in an outpatient procedure, and none had to stay overnight because of complications.

The general recommendation was to refrain from work for 6 weeks, but people with a physically light job were able to return to work after 2 weeks.

#### Failure rates

In the SBG, five patients underwent a re-operation within the 2-year follow-up period. The causes were pain (*n* = 2), loose body (three times in the same patient) and meniscal injury (*n* = 2). The corresponding number in the DBG was six patients, pain (*n* = 1), meniscal and chondral injury (*n* = 1), chondral injury (*n* = 1), swelling (*n* = 1), extension deficit (*n* = 1) and arthroscopic evaluation (*n* = 1) (Table 1).

In the present study, post-operative appointments were made at the orthopaedic ward after 4–6 weeks, seeing only the surgeon, and at 6 and 24 months after surgery, seeing both the surgeon and the physiotherapist. The clinical examination included range of motion (ROM), knee laxity measurements, functional tests commonly used to evaluate ACL-reconstructed patients, as well as radiographic examination. The routine is one post-operative visit to the surgeon after 4–6 weeks and one to the physiotherapist after 6 months, but, as these patients were included in a randomised clinical trial study (RCT) comparing the two surgical methods, visits to the surgeon at six and 24 months were included, as well as the X-ray examination.

### Post-operative rehabilitation programme

A referral was given to the patients when leaving the hospital with the recommendation to contact their local physiotherapist and start rehabilitation seven to 10 days post-operatively [1]. All the patients were rehabilitated according to the same guidelines.

#### Data extraction

All patient reports, as well as surgical reports, were reviewed by two persons independently. Disagreement or uncertainty between two reviewers was resolved by consensus or after discussion with a third party, one of the surgeons.

### Outcome measures

#### Health-related quality of life and quality-adjusted life years

##### EQ-5D

The EQ-5D is a generic instrument for measuring overall health-related quality of life based on five dimensions (mobility, self-care, usual activities, pain/discomfort and depression/anxiety) and including three levels (none, moderate and severe problems) of answers and a rating scale. In the present study, the UK tariff values of scale between −0.59 and 1 have been used. These social tariff values (elicited health state valuations from a representative sample of the UK health population) have previously been published [12]. A summary index between 1 (full health), 0 (death) and values below 0 corresponding to worse than death has been calculated [8, 34]. A difference of 0.07 or more is regarded as clinically relevant [39]. It was intended to be used for economic analysis (cost-utility analysis) and to make it possible to calculate the cost per QALY [8]. Brazier et al. [6] evaluated the EQ-5D in a group of patients with osteoarthritis of the knee and concluded that it could be used for economic evaluations of surgery.

#### QALY

The quality of life weighting was then used to calculate QALYs. This measurement combines years of life with quality of life, so that the QALY, as a result of a treatment, can consist of increasing life expectancy and/or increased quality of life. QALY calculations (here using the EQ-5D) were made at individual level, reflecting the change from baseline to 2 years, assuming a linear change in QoL between the two measurements. QALY was calculated by using the area-under-the-curve approach.

$${\text{QALY}} = \left( {\left( {{\text{EQ5D baseline }} + {\text{EQ5D }}2 {\text{years}}} \right)/2} \right) \times 2$$

### Costs

The cost in the current study consisted of direct and indirect costs (production loss). The direct costs were calculated from resources used in specialised outpatient care. These costs included all fixed costs, i.e. administration and other overhead costs, staff salaries and accommodation at recovery, as well as individual costs, such as examination, surgery (operating room (OpR), anaesthesia and material), post-operative visits, rehabilitation, laboratory tests or imaging. All costs were collected from the hospitals’ administrative records registered at individual level (e.g. hospitalisation), including fixed cost for the hospital stay and specific resources used during the treatment.

The indirect costs are costs related to reduce work ability for health reasons. The human capital method was used in this study, estimating the potential production loss, based on the assumption that earnings reflect productivity [13]. Using this method, the daily production loss on an individual basis was calculated and consisted of each individual´s number of days of sick leave, obtained from the National Social Insurance Board’s (NSIB) central register (http://www.forsakringskassan.se/privatpers). All sick leave periods longer than 14 days and all disability pension payments are administered and registered by the NSIB. Statistics Sweden (http://www.scb.se/Pages/List____250619.aspx) supplied the information relating to the national mean monthly salary for males and females presented for defined professions. As 15% and 24%, respectively, were students, the imputation of missing data for indirect costs was performed. Furthermore, the average salary (for defined professions among the study participants) from Statistics Sweden (http://www.scb.se) and payroll taxes (SEK 1334 per day of absence) were used to calculate the cost of productivity losses.

Other costs associated with cruciate ligament damage, such as travelling to and from treatment, are not valued.

All the costs were obtained and analysed retrospectively. The costs are presented in euros, using the 2010 average exchange rate (Euro 1 = SEK 9.55).

#### Cost-utility analysis

Cost-utility analysis (CUA) refers to a particular form of cost-effectiveness analysis where the outcome is measured in terms of QALY gained. This can be compared with the mean total cost (direct + indirect costs) of the programme to determine the cost per QALY gained.

In this study, both QALY and costs are discounted by 3%.

$${\text{Incremental cost - effectiveness ratio }}\left( {\text{ICER}} \right) = \frac{{\Delta {\text{Cost(Cost}}_{\text{DBG}} - {\text{Cost}}_{\text{SBG}} )}}{{\Delta {\text{Effect }}\left( {{\text{QALY}}_{\text{DBG}} {-}{\text{QALY}}_{\text{SBG}} } \right)}}$$

The ICER can be interpreted as the extra cost of obtaining one extra QALY and allows comparisons between interventions in all areas of health care, not necessarily comparable programmes [10]. The results of the analysis are able to demonstrate whether the DB technique yields higher or reduced costs, reduced or improved utility in comparison with the SB technique and are presented in a so-called cost-effectiveness plane [13].

#### ICER uncertainty

The cost-effectiveness plane was used to visualise the uncertainty surrounding the mean ICER point estimate. The plane displays the incremental costs on the *y*-axis and the incremental effects on the x-axis and is divided into four quadrants: north-east (NE), south-east (SE), south-west (SW) and north-west (NW) [15]. If the ICERs are plotted in the NE quadrant, the DB technique yields higher costs and an improved effect and a willingness-to-pay (WTP) threshold would therefore be necessary, if the DB technique were implemented. Conversely, if ICERs are plotted in the SE quadrant, the DB technique both saves costs and has an improved effect; the DB technique is therefore the dominant strategy. If the ICERs are located in the SW quadrant, the DB technique reduces costs, but the effect decreases. Finally, if the ICERs are placed in the NW quadrant, the DB technique is both more expensive and reduces the effect, which explains why the existing treatment, the SB technique, clearly dominates.

The cost-effectiveness acceptability curve (CEAC) is constructed so that it shows the proportion of simulations that are deemed cost-effective, given different threshold values for cost-effectiveness. It is the proportion of simulations to the right of a budget line with the slope corresponding to the threshold value in the figures. The CEAC illustrates the probability that the DB technique is cost-effective compared with the SB technique at different willingness-to-pay thresholds [13]. In this analysis, the maximum willingness to pay was set at a level of Euro 50,000 (SEK 500,000), based on the Swedish National Board of Health and Welfare (https://www.socialstyrelsen.se/SiteCollectionDocuments/metodbeskrivning-nationella-riktlinjer.pdf).

#### Sensitivity analysis

To assess the strength of the result, an average salary from Statistics Sweden (http://www.scb.se) and payroll taxes were used to estimate the cost of productivity loss (SEK 1400 (=Euro 147) per day of absence).

As a sensitivity analysis, both costs and effects are discounted at 5 and 0% (no discount) and costs are discounted at 3%, but no discount is made for effects. A health-care perspective using the direct costs is also presented.

### Statistical analysis

Sample size calculation was based on the pivot shift test with one degree of difference, a power of 0.85 and a level of significance of 0.05, which gives 38 patients in each group.

Mean (SD) and median (range) values are presented when applicable. For comparisons of dichotomous variables between the groups, the Chi-square test was used. In terms of comparison between the groups of continuous variables, a *t* test was used, while for discrete variables the Mann–Whitney *U* test was used. A *p*-value of ≤ 0.05 was considered statistically significant. Effect size was calculated as the difference between pre-operative values and post-operative values.

By using bootstrapping, we wanted to assess the statistical uncertainty associated with the ICER. We simulate new ICERs as if they were single mean estimates from the trial by building up an empirical sampling distribution (based on the original data) in order to make inferences about the ICER. The number of units is the same as the number of patients in the analysis and the CI is based on 1000 such samplings. The bootstrapping analysis was performed using the SAS 9.2 computer program, Cary, NC.

### Ethics

Eligible patients were informed about the study and gave their signed informed consent. The Regional Ethical Review Boards at the University of Gothenburg approved the study, (2011-07-25) Dnr 162-11, and permission was also obtained from the heads of the individual clinics.

## Results

Pre-operatively, the groups were comparable in terms of demographic data (Table 1).

Descriptive data relating to the total intervention procedure are presented in Tables 2 and 3. The sensitivity analysis is presented in Table 4.

**Table 2:** Clinical resource use and days of sick leave

	Double bundle (DB) *n* = 53	Single bundle (SB) *n* = 50	*P* value SB versus DB	Source
Operating room (OpR) time				
Mean (SD)	68.4 (12.7)	56.8 (16.5)	**0.001**	VGR medical record
Median (range)	70 (41–92)	55.5 (26–90)		
Number of post-op visits to the surgeon				
Mean (SD)	3.3 (0.9)	3.4 (0.9)	n.s.	VGR medical record
Median (range)	3 (2–7)	3 (2–6)		
Consulting phone calls to surgeons				
Mean (SD)	0.43 (1.22)	0.34 (0.69)	n.s.	VGR medical record
Median (range)	0 (0–8)	0 (0–3)		
Consulting phone calls to nurse on the orthopaedic ward				
Mean (SD)	0.08 (0.27)	0.20 (0.73)	n.s.	VGR medical record
Median (range)	0 (0–1)	0 (0–4)		
Associated injuries addressed at the time of the index operation or during the follow-up period				
Meniscal (medial and/or lateral)	15 (28%)	18 (36%)	n.s.	VGR medical record
Meniscal and chondral	14 (26%)	13 (26%)		
Chondral	6 (11%)	7 (14%)		
None	18 (34%)	12 (24%)		
Registered complications				
None	48 (90.6%)	45 (90%)	n.s.	VGR medical record
Bleeding	2 (3.8%)	1 (2%)		
Extension deficit	1 (1.9%)	–		
Fever	1 (1.9%)	1 (2%)		
Infection	–	1 (2%)		
Locking	1 (1.9%)	1 (2%)		
Pain/swelling	–	1 (2%)		
Re-operations yes/no	6/47	5/45	n.s.	VGR medical record
Meniscal/cartilage	3 (6%)	3 (6%)		
Loose bodies		1 (2%)		
Notchplasty	1 (2%)	1 (2%)		
Visits to physiotherapist				
Mean (SD)	18.9 (20.7)	19.8 (18.4)	n.s.	PT^a^
Median (range)	13 (0-88)	15.5 (0-68)		
Sick leave (total days)				
Mean (SD)	64.6 (87.5)	80.2 (82.5)	n.s.	N S I B
Median (range)	32.75 (5–442)	45.0 (5–284)		
Missing values	9	13		

Bold value represent *p*-value of ≤ 0.05 was considered statistically significant

*VGR* Västra Götaland Region, Sweden, *NSIB* National Social Insurance Board

^a^ Personal contact with each physiotherapy department rehabilitating ACL injuries

**Table 3:** Mean cost per resource use variable and difference

	Mean cost per patient (Euro)/DB *n* = 53	Mean cost per patient (Euro)/SB *n* = 50	Difference in mean costs (Euro)	*P* value SB versus DB	Source
Costs OpR^a^ surgery					
Mean (SD)	846 (757)	651 (633)	195	**0.019**	VGR
Median (range)	453 (185–3258)	351 (146–3289)			
Cost OpR^a^ anaesthesia					
Mean (SD)	1077 (653)	794 (505)	283	**0.041**	VGR
Median (range)	1077 (194–2064)	794 (204–1790)			
Costs ReR^b^					
Mean (SD)	119 (33)	113 (33)	6	n.s.	VGR
Median (range)	119 (78–207)	113 (61–175)			
Other costs^c^					
Mean (SD)	876 (16.1)	892 (13.1)	16	n.s.	VGR
Median (range)	876 (851–920)	892 (869–920)			
Costs rehabilitation (PT)^f^					
Mean (SD)	1, 895 (2078)	1980 (1839)	−85	n.s.	
Median (range)	1307 (0–8818)	1553 (0–6814)			
Total direct costs (sum)					
Mean (SD)	4813 (2274)	4430 (2334)	469	n.s.	VGR
Median (range)	4463 (1916–11,002)	3903 (1579–10,738)			
Monthly salary (Euro)					
Mean (SD)	3025 (842)	2785 (597)			SCB
Median (range)	2796 (2095–6387)	2660 (2105–5330)	240	n.s	
Missing values	9	14			
Production loss^d^ (Euro)					
Mean (SD)	8145 (9373)	9063 (7993)	−918	n.s.	N S I B
Median (range)	5769 (459–50,079)	8632 (560–33,480)			
Total costs (Euro)	12,958	13,493			
Total costs discounted (3%)^e^	12,569	13,088			
Mean (SD)	13,199 (9910)	13,762 (8526)	−563	n.s.	
Median (range)	11,180 (3194–55,476)	11,823 (2588–36,478)			

Bold value represent *p*-value of ≤ 0.05 was considered statistically significant

*DB* double-bundle technique, *SB* single-bundle technique, *VGR* Västra Götaland region, *NSIB* National Social Insurance Board, Sweden, *SCB* Statistics Sweden

^a^ Operating room, including cost of bundle, 260 euro (SBG) and 548 euro (DBG)

^b^ Recovery room

^**c**^ Other costs include an overall cost for peripheral costs such as post-operative visits, consulting time OpR. Anaesthesia. ReR and post-op visits

^d^ Monthly salary (SCB) × 1.3142 (social contributions)/30 × days of sick leave, based on imputed data

^e^ Sum of total direct costs + production loss

^f^ Physiotherapy

**Table 4:** Sensitivity analysis and production loss based on average of wages from Statistics Sweden

	Mean cost per patient (Euro)/DB *n* = 53	Mean cost per patient (Euro)/SB *n* = 50	Difference in mean costs (Euro)	*P* value SB versus DB	Source
Production loss^c^					
Mean (SD)^a^	8145 (9373)	9063 (7993)			
Mean (SD)^b^	9329 (11,666)	10,944 (10,454)	−1615	n.s	NSIB
Median (range)	7147 (733–64,796)	8632 (733–41,634)			
Total costs discounted (3%)					
Mean (SD)^a^	12,569	13,088			
Mean (SD)^b^	13,718 (11,505)	14,913 (10,295)	−1195	n.s	
Median (range)	10,484 (3128–66,633)	12,043 (2475–42,275)			

*NSIB* National Social Insurance Board, based on average of wages from Statistics Sweden

^a^ Based on the average salary of included professions

^b^ Based on average salary according to Statistics Sweden

^c^ Direct and indirect costs

The groups were comparable pre-operatively and at follow-up in terms of HRQoL (Table 5).

**Table 5:** EQ-5D and QALY

	Double bundle (DB) *n* = 53	Single bundle (SB) *n* = 50	*P* value SB versus DB
EQ-5D pre-op			
Mean (SD)	0.639 (0.29)	0.639 (0.24)	n.s.
Median (range)	0.727 (−0.077 to 1.00)	0.691 (0–1.00)	
EQ-5D 2 years Fp*			
Mean (SD)	0.839 (0.22)	0.849 (0.19)	n.s.
Median (range)	0.848 (0.088–1.00)	0.822 (0.088–1.00)	
Missing value	2	2	
Difference between pre-op and 2 years	0.201 (0.31)	0.212 (0.27)	n.s.
QALY			
Mean (SD)	1.48 (0.41)	1.49 (0.34)	n.s.
Median (range)	1.59 (0.29–2.0)	1.52 (0.12–2.0)	
Missing values	2	2	
QALY discounted 3%			
Mean (SD)	1.43 (0.40)	1.44 (0.33)	n.s.
Median (range)	1.54 (0.29–1.94)	1.48 (0.11–1.94)	
Missing values	2	2	

* *P* < 0.001, comparison between pre-operative and 2-year follow-up values within the groups

**Fig. 2:**
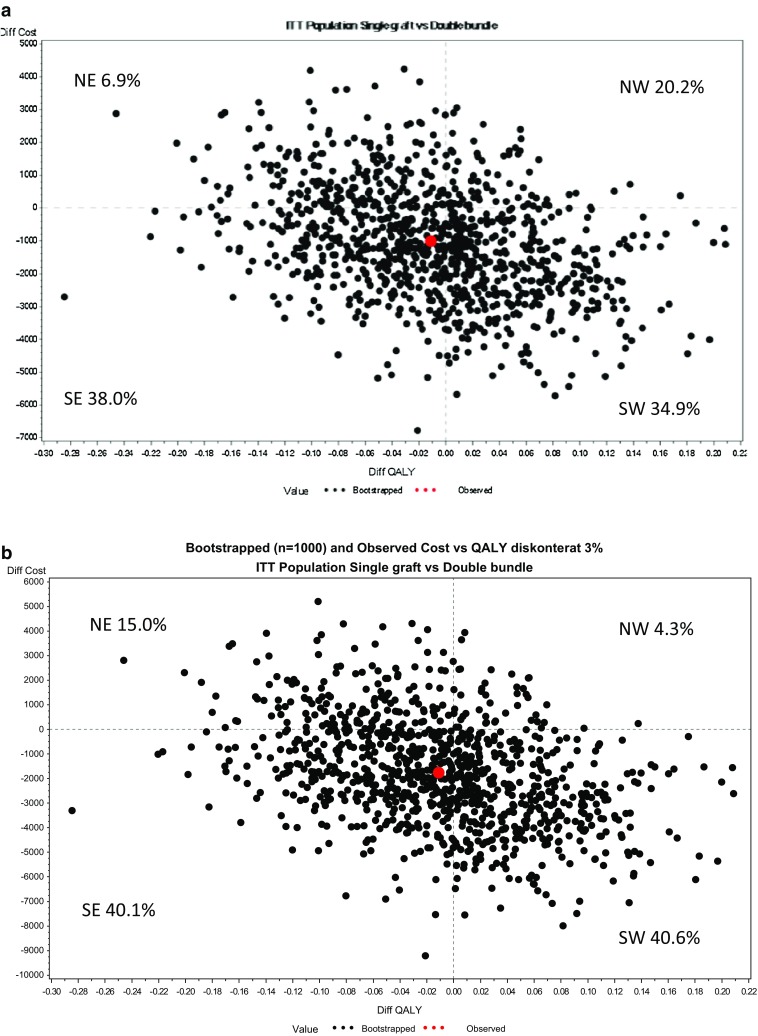
**a** Incremental cost-effectiveness ratio (ICER) of DB versus SB for patients with ACL injury. The costs are based on the average salary of included professions. **b** The incremental cost-effectiveness ratio (ICER) of DB versus SB for patients with ACL injury. ICER based on average salary according to Statistics Sweden

The ICER points (based on the average salary for defined professions among the study participants) were distributed in each of the four quadrants, where 20.2% were located in the NW quadrant, 34.9% in the SW quadrant, 38.0% in the SE quadrant and 6.9% in the NE quadrant. In other words, the ICERS are spread out over all the quadrants (Fig. 2a).

**Fig. 3:**
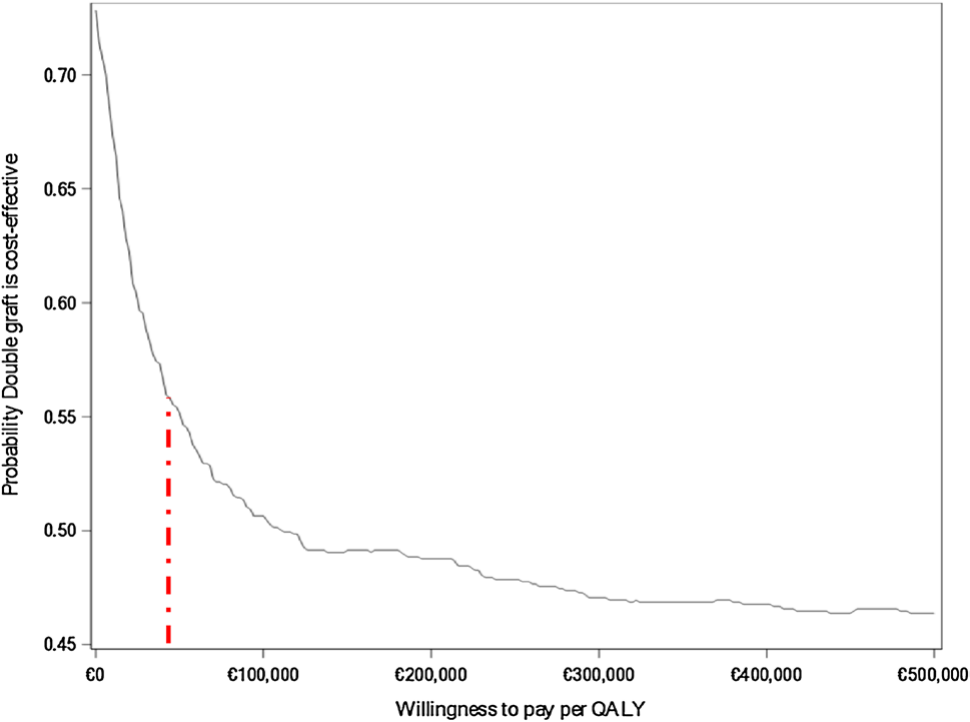
Cost-effectiveness acceptability curve (CEAC), based on average salary of included professions. The red dotted line shows that the maximum willingness to pay was set at a level of €50,000 (SEK 500,000) based on the Swedish National Board of Health Care

The CEAC (Fig. 3) is based on the average salary for defined professions and means that there is a 50% probability of the DB technique being cost-effective at a willingness-to-pay threshold of Euro 50,000 with great uncertainty in the ICER estimates.

### Sensitivity analysis

The ICER point (based on average salary for defined professions) was distributed in each of the four quadrants, where 20.2% was located in the NW quadrant, 34.9% in the SW quadrant, 38.0% in the SE quadrant and 6.9% in the NE quadrant (Fig. 2a).

The CEAC (Fig. 3) based on average salary for defined professions there is a 50 probability of DBG being cost-effective at a threshold of willingness to pay of Euro 53,000, however with a great uncertainty in the ICER estimates.

The total mean cost for DBG and SBG, when using the average salary from Statistics Sweden, was Euro 13,718 and Euro 14,913, respectively (Table 4), which is slightly higher costs in comparison with the national mean monthly salary for males and females presented for defined professions (Table 3). The ICERs based on average salary (Fig. 2b) showed that 40.6% was located in the SW, 40.1% in the SE, 4.3% in the NW and 15% in the NE quadrant. Here the CEAC showed that around 55 probability of DB being cost-effective at a willingness-to-pay threshold of Euro 53,000.

The discount rate 0, 3 and 5% for costs and QALYs is presented in Table 6.

**Table 6:** Sensitivity analysis and discount rate 0, 3 and 5% for costs and QALYs

Discount rate	Double bundle (DB) *n* = 53	Single bundle (SB) *n* = 50	Difference	Cost-effectiveness plane
*Societal perspective*				
0% total costs	12,958	13,439	−481	Cheaper/worse
0% QALY	1.48	1.49	−0.01	
5% total costs	12,310	12,767	−457	Cheaper/worse
5% QALY	1.41	1.42	−0.01	
3% total costs	12,569	13,088	−519	Cheaper/worse
0% QALY	1.48	1.49	−0.01	
*Health-care perspective*				
3% direct costs	4717	4297	420	More expensive/worse
3% QALY	1.43	1.44	−0.01	

## Discussion

The principal findings in the present study are that the double-bundle technique is more expensive, as it takes longer in the operating room and requires more materials (direct costs *p* = 0.005), which means that the DB is more expensive from a health-care perspective. However, from a societal perspective there was no statistically significant difference between the groups receiving SBG and DBG, due to lower costs for production loss among patients in the DB group. This was confirmed when analysing both average salary for defined professions among the study participants and for national mean monthly salary (Fig. 2a, b). There was no difference between the groups in terms of QALYs.

### Single-bundle versus double-bundle technique

In a recent systematic review by Mascarenhas et al. [29] analysing nine meta-analyses, the conclusion was that the double-bundle technique provided better post-operative knee stability, as measured with the KT 1000 arthrometer and pivot shift. This is also in line with the present study [1]. The 5-year follow-up of the present study has recently been published, and no statistical differences could be found between the groups in terms of clinical outcome or the presence of osteoarthritis [24].

However, the development of OA may take longer than 5 years to be radiographically visible. From a societal perspective, the future costs may include the consequences of the development of OA, such as sick leave and the need for a total knee arthroplasty. Today, according to the Swedish ACL Register, the use of the DB technique accounted for fewer than 1% of all the operations performed in 2015 [17]. The main reason from the health-care perspective for not implementing the DB technique is higher direct costs, with a clinical outcome comparable to that produced by the SB technique.

In a meta-analysis, Li et al. [28] pooled data from seven studies and found no evidence of a significant difference in complication rate between the single- and double-bundle techniques. This is in line with the present study. No complications were registered in 90% of the patients, both groups, respectively. The low complication rate is also seen in the post-operative visits and consulting phone calls to the surgeon or nurse on the orthopaedic ward within standard care. No re-ruptures or revision surgery was registered during the follow-up period in the present study [1].

### Total costs

#### Direct costs

Orthopaedics, especially sporting injuries, are often given as an explanation of the increased costs within the health-care system and orthopaedic surgeons have to justify their choice of procedure [5, 20]. These costs should be compared with the reduction in hospital stay or outpatient surgery and earlier, accelerated rehabilitation. This results in an improved functional outcome for the patient and hopefully a smaller burden on society [11].

The cost of ACL reconstructions, as well as other orthopaedic sports medicine injuries, is part of the entire health-care system and is charged to the orthopaedic department, along with all the other orthopaedic diagnoses. Nwachukwu et al. [32] evaluated the cost-effectiveness analyses in orthopaedic sports medicine literature and suggested that ACL reconstruction was cost-effective. On the other hand, a recent study published by Kiadiliri et al. [26] found no statistically significant difference in economic value in terms of early ACL reconstruction compared with rehabilitation and ACL reconstruction when needed. They may improve the outcome, but at greater expense and with higher administrative costs, plus lapses in quality [2]. An ACL injury is a serious knee injury, and it is more common in young athletes performing highly demanding sports. They are often very eager to have the ‘best’ (= newest) treatment, resulting in a rapid return to their previous level of sporting activity. At a time at which new technology and techniques are easily spread and popularised through social media, there is a need critically to evaluate not only the clinical results, but also the costs, before any implementation in clinical practice. Saltzman et al. conducted an economic analysis of ACL reconstruction comparing different surgical techniques, including the type of grafts, and concluded that the single-bundle technique offered greater economic value compared with the double-bundle technique. This is in line with Brophy et al. [9] who concluded that the double-bundle technique should not be accepted as the new standard technique until evidence of improved clinical outcome is presented. On the other hand, only the study by Paxton et al. used cost-utility analysis and ICER, showing that the double-bundle technique is a cost-effective procedure [33, 35]. According to the ACL Register, in 2014, only 28 ACL reconstructions of 3430 were performed using the double-bundle technique [16].

#### Production loss (indirect costs)

The total days of sick leave differed between the two groups, 80 days in the SBG and 65 days in the DBG, but this difference was not statistically significant. The median difference of 15 min in time in the operating room may not result in post-operative discomfort and longer sick leave (production loss). The difference in sick leave could reflect differences in occupation between the two groups, prolonging the return to work, irrespective of the type of reconstruction technique used. There were, for example, more self-employed people in the DBG (30 vs 14%), which could suggest a greater eagerness to resume work. The larger number of days off work in the SBG might, however, randomly also reflect the fact that working ability and sick leave are highly subjective measurements with a large impact on costs in a study like this [21]. There are missing data on sick leave in 22 patients (13 in the SBG and nine in the DBG), and this might be due to unemployment, being student or being left out of the insurance system for other reasons. However, imputed data were used in the analysis. In this study, only post-operative sick leave periods longer than 14 days and all payments are included. The difference in health-care organisation and insurance system between countries must also be taken into account.

In recent years, focusing on rehabilitation programmes and the criteria for knee function has been a topic of great interest, as the return to competitive sport is relatively low [23]. However, the primary objective, after all, is to be able to return to work. Even though the individual has a legal right to compensation due to lost income through the Swedish social insurance system, this does not cover the whole loss of income. Heavy industrial workers and craftsmen have to regain full muscle strength, as well as health-related quality of life.

The health-related quality of life result, as measured with the EQ-5D, was in line with data from the Swedish National ACL database, comparing the single-bundle and double-bundle technique [4], but lower compared with the general Swedish population [36].

As the DB trial was primarily designed to detect a difference of one degree in the pivot shift test, the number of enrolled subjects may have been too low to detect a difference in QALY during the trial period. The baseline analysis conducted prior to the CUA did not detect any statistically significant differences in baseline utility. Further, the collection of QALYs in the DB trial could have been improved, as the EQ-5D score was only measured at baseline and at the 2-year follow-up. In the light of the fact that patients after ACL reconstruction are not re-admitted to the hospital due to re-injury to the ACL, it was not necessary to include further EQ-5D score measurements in the trial and the follow-up period, as this would not have increased the accuracy of the results.

#### QALY

In the present study, the QALY for the single-bundle group and double-bundle group was 1.44 and 1.43, respectively. This means that there was no gain in QALYs for the DB group (−0.01). Performing a cost-effectiveness comparison of different types of graft, Genuario et al. [19] reported a QALY of between 0.904 and 0.912, while Farshad et al. [14] compared conservative treatment with ACL reconstruction and reported a QALY of 0.66.

The gain in QALY is also higher (0.212 and 0.201, respectively) compared with the cost-utility analysis conducted on the Multicentre Orthopedics Outcomes Network (MOON) database (0.18) [30].

The cost-effectiveness plane, where the 1000 bootstrap replicates of the ICER were spread out of over the quadrants, demonstrated that the DB cost almost as much as the SB and had the same effect as the SB (Fig. 2). The sensitivity analysis revealed marginal/no differences between the discount rates of 0 and 5% for costs and QALYs. From a health-care perspective, the DB technique cannot be recommended. However, from the patients’ perspective, the physician should consider individual treatment based on who the patient is and what his/her expectations and requirements are [25]. Other aspects to take into consideration are the preferences, experiences and skill of the orthopaedic surgeons when choosing the technique.

### Strengths and limitations

One strength of the present study is the randomised design and the follow-up rate of 98%. The clinical results are also in line with those in previous studies. It is common for the sample size of a trial to be based on the primary clinical outcome, and in this study, the sample size was calculated on the difference in pivot shift and not on differences in health-related quality of life. This is a major limitation, as, in a calculation based on the difference in sick leave or total costs, the power will be low. However, comparing costs, as in the present study, takes place alongside an analysis based on a clinical randomised trial. Moreover, the change in the EQ-5D over time is based on two measurement points (pre-operatively and at the 2-year follow-up), assuming a linear function using an area-under-the-curve approach. The first 2 weeks of sick leave are paid by the employer, while sick leave periods longer than 14 days are administered and registered by the NSIB. As the recommended period of sick leave after an ACL reconstruction is at least 6 weeks, the total costs including the employers’ contribution may have been higher, but the difference between groups would be the same. Another limitation is that the direct costs at a university hospital may have been higher than those at a county hospital. There was, however, no intention to compare the two hospitals. The fact that the cost analyses are dependent on the health-care cost and policy of the specific country, thereby limiting the external validity, must also be taken into consideration.

### Clinical perspective

The results of the present study give the physician the possibility to choose an individualised treatment based on age, gender, symptoms and the size of the native ACL insertion site, but also have a detailed discussion with the patient relating to expectations and requirements [25], such as rehabilitation intensity and demands relating to activity level, in terms of both sports activity level and work [3]. In the long term, this will hopefully be more cost-effective for both the patient and society.

## Conclusion

The double-bundle technique is more expensive than the single-bundle technique, as it takes longer in the operating room and requires more materials. On the other hand, no difference was found in total costs, due to lower costs for production losses among patients in the DB group. Furthermore, no difference in QALYs was seen between the groups.

## Abbreviations

ACL: Anterior cruciate ligament
CEAC: Cost-effectiveness acceptability curve
CUA: Cost-utility analysis
DB: Double-bundle technique
DBG: Double-bundle group
ICER: Incremental cost-effectiveness ratio
KOOS: Knee injury and osteoarthritis and outcome score
NE: North-east
NW: North-west
NSIB: National social insurance board
OA: Osteoarthritis
QALY: Quality-adjusted life year
RCT: Randomised clinical trial
ROM: Range of motion
SB: Single-bundle technique
SBG: Single-bundle group
SD: Standard deviation
SE: South-east
SW: South-west
